# Supplementary material for: Retinoic Acid Signaling Is Associated with Cell Proliferation, Muscle Cell Dedifferentiation, and Overall Rudiment Size during Intestinal Regeneration in the Sea Cucumber, Holothuria glaberrima
Source: Biomolecules. 2019 Dec 13;9(12):873. doi: 10.3390/biom9120873 (PMC6995554; doi:10.3390/biom9120873)
Supplement: Supplementary file 1 [file biomolecules-09-00873-s001.pdf]

**Supplementary Material**

Table S1. Protein sequences employed for the multiple sequence alignments and/or phylogenetic analyses.

Short-chain dehydrogenase reductase

| Nickname | Organism scientific name | NIH-GenBank accession no. |
|---|---|---|
| acorn worm.7 | *Saccoglossus kowalevskii* | XP_006817028.1 |
| apis.7 | *Apis mellifera* | XP_394428.1 |
| bristle worm.7 | *Capitella teleta* | ELT96525.1 |
| canis.7 | *Canis lupus familiaris* | XP_537465.3 |
| ciona.7 | *Ciona intestinalis* | XP_002123111 |
| criter.7 | *Cricetulus griseus* | XP_007618527.1 |
| drosophila.7 | *Drosophila melanogaster* | Q9Y140.1 |
| gallus.7 | *Gallus gallus* | XP_421423.2 |
| homo.7 | *Homo sapiens* | NP_057113.1 |
| lancelet.7 | *Branchiostoma belcheri* | XP_019640821.1 |
| mus.7 | *Mus musculus* | NP_079798.2 |
| oyster.7 | *Crassostrea gigas* | XP_011444744.1 |
| rattus.7 | *Rattus norvegicus* | NP_001258323.1 |
| scallop.7 | *Mizuhopecten yessoensis* | XP_021379944.1 |
| sea cucumber.7 | *Holothuria glaberrima* | MN124283 |
| tilapia.7 | *Oreochromis niloticus* | XP_019204754.1 |
| urchin.7 | *Strongylocentrotus purpuratus* | XP_790920.3 |
| xenopus.7 | *Xenopus laevis* | NP_001085784.2 |

Other aldehyde dehydrogenases/reductases from the *Strongylocentrotus purpuratus* genome

| Nickname | Gene | NIH-GenBank accession no. |
|---|---|---|
| urchin.1 | SDR 1 | XP_797581.2 |
| urchin.4 | SDR 4 | XP_001200926.2 |
| urchin.11 | SDR 11 | XP_011679965.1 |
| urchin.12 | SDR 12 | XP_793972.3 |
| urchin.42E | SDR 42E1 | XP_011676811.1 |
| urchin.rd7 | retinol dehydrogenase 7 | XP_786807.3 |
| urchin.rd8 | retinol dehydrogenase 8 | XP_011671426 |
| urchin.rd11 | retinol dehydrogenase 11 | XP_011661792.1 |
| urchin.rd12 | retinol dehydrogenase 12 | XP_790111.3 |
| urchin.Xc | SDR chromosome X | XP_011682059.1 |

Aldehyde dehydrogenase family 8A1

| Nickname | Organism scientific name | NIH-GenBank accession no. |
|---|---|---|
| canis.8 | *Canis lupus familiaris* | XP_533415.2 |
| gallus.8 | *Gallus gallus* | XP_419732.1 |
| hamster.8 | *Cricetulus griseus* | XP_016835510.1 |
| homo.8 | *Homo sapiens* | NP_072090.1 |
| horseshoe crab.8 | *Limulus polyphemus* | XP_0222564211 |
| hydra.8 | *Hydra vulgaris* | XP_004206718.1 |
| lancelet.8 | *Branchiostoma belcheri* | XP_019632799.1 |
| mite bee.8 | *Varroa destructor* | XP_022663935.2 |
| mus.8 | *Mus musculus* | NP_848828.1 |
| oyster.8 | *Crassostrea gigas* | XP_011454749.1 |
| rattus.8 | *Rattus norvegicus* | NP_001178017.2 |
| sea cucumber.8 | *Holothuria glaberrima* | MN124284 |
| sea squirt.8 | *Ciona intestinalis* | XP_002126639.1 |
| sea slug.8 | *Aplysia californica* | XP_005105733.1 |
| spider.8 | *Parasteatoda tepidariorum* | XP_015920063.1 |
| starfish.8 | *Acanthaster planci* | XP_022088838 |
| tilapia.8 | *Oreochromis niloticus* | XP_003440690.1 |
| urchin.8 | *Strongylocentrotus purpuratus* | XP_011673512.1 |
| urochord.8 | *Oikopleura dioica* | ANP24208.1 |
| xenopus.8 | *Xenopus tropicalis* | XP_002935852.1 |

Other aldehyde dehydrogenases from the *Mus musculus* and *Strongylocentrotus purpuratus* genomes

| Nickname | Gene | NIH-GenBank accession no. |
|---|---|---|
| mus.R1 | retinal dehydrogenase 1 | NP_038495.2 |
| mus.R2 | retinal dehydrogenase 2 | NP_033048.2 |
| mus.R3 | retinal dehydrogenase 3 | AAF86980 .1 |
| urchin.9A1 | ALDH 9A1 | XP_011676578.1 |
| urchin.16A1 | ALDH 16A1 | XP_797815.1 |
| urchin.fatty | fatty aldehyde dehydrogenase | XP_011678668.1 |
| urchin.mit | aldehyde dehydrogenase mitochondrial | XP_011675858.1 |
| urchin.NADP | aldehyde dehydrogenase NADP | XP_011679535 .1 |
| urchin.rd12 | retinol dehydrogenase 12 | XP_790111.3 |
| urchin.Xc | SDR chromosome X | XP_011682059.1 |
